# Impacts of the Invasive Seaweed *Asparagopsis armata* Exudate on Energetic Metabolism of Rock Pool Invertebrates

**DOI:** 10.3390/toxins13010015

**Published:** 2020-12-25

**Authors:** Carla O. Silva, Sara C. Novais, Amadeu M. V. M. Soares, Carlos Barata, Marco F. L. Lemos

**Affiliations:** 1 MARE—Marine and Environmental Sciences Centre, ESTM, Polytechnic of Leiria, 2520-641 Peniche, Portugal; carla.o.silva@ipleiria.pt (C.O.S.); sara.novais@ipleiria.pt (S.C.N.); 2 Department of Biology and CESAM (Centre for Environmental and Marine Studies), University of Aveiro, 3810-193 Aveiro, Portugal; asoares@ua.pt; 3 Environmental Chemistry Department, IDAEA-CSIC, Jordi Girona 18-26, 08034 Barcelona, Spain; cbmqam@cid.csic.es

**Keywords:** ecotoxicology, invasive species, *Gibbula umbilicalis*, *Palaemon elegans*, tidal pools

## Abstract

The marine red algae *Asparagopsis armata* is an invasive species gaining competitive advantage by releasing large amounts of toxic compounds to the surrounding invaded area. The main objective of this study was to evaluate the effects of this invasive seaweed on marine invertebrates by exposing the common prawn *Palaemon elegans* and the marine snail *Gibbula umbilicalis* to the exudate of this seaweed. The seaweed was collected and placed in a tank for 12 h in the dark in a 1:10 ratio. Afterwards the seawater medium containing the released secondary metabolites was collected for further testing. Lethal and sublethal effects of *A. armata* were investigated. Biochemical biomarker responses associated with energy metabolism (lactate dehydrogenase, LDH; electron transport system activity, ETS; lipid, protein and carbohydrate content) were analysed. The biomarker responses showed physiological status impairment of invertebrates after exposure to low concentrations of this algal exudate. The highest concentrations of exudate significantly increased lipid content in both organisms. In the shrimp, protein content, ETS, and LDH were also significantly increased. By contrast, these parameters were significantly decreased in *G. umbilicalis*. A behavioural impairment was also observed in *G. umbilicalis* exposed to *A. armata* exudate, reducing feeding consumption. These results represent an important step in the research of natural toxic exudates released to the environment and prospective effects of this seaweed in invaded communities under increasing global change scenarios.

## 1. Introduction

Rapid globalization and increasing trends of trade and travel have accelerated marine biological invasions by transporting species to areas outside their native range. These non-indigenous species (NIS) are considered invasive, and once they are established, can spread rapidly and proliferate and thereby dominate the new habitat without the direct intervention of humans [1]. *Asparagopsis armata* Harvey, 1855 (Bonnemaisoniales, Rhodophyta) is a red seaweed native to Western Australia, and nowadays is distributed throughout Europe in the Atlantic and Mediterranean basin, where it is highly invasive [2]. This seaweed possesses chemical defence mechanisms that are critical for its invasiveness, based on the synthesis and storage of an array of secondary metabolites, which include more than 100 halogenated compounds such as haloforms, haloacids, and haloketones [3]. These volatile halogenated hydrocarbons, containing one to four carbons, are known antifeedant and cytotoxic compounds, among other properties (reviewed in Pinteus et al. [4]). The pungent aroma of this alga is attributed to an essential oil that is composed mainly of bromoform with smaller amounts of other bromine-, chlorine-, and iodine-containing methane, ethane, ethanol, acetaldehydes, acetones, 2-acetoxypropanes, propenes, epoxypropanes, acroleins, and butenones, stored in vacuoles within gland cells [5]. These potent biological effects of these compounds can induce significant changes in terms of native community composition [6], including in rock pools [7], favouring *A. armata* in a given niche. This aggressive invasive behaviour has placed this species in the 100 “worst invasives” in the Mediterranean [8], and its rate of invasion is steadily increasing [9] threatening native biodiversity [10]. Due to the enclosed environment during low-tide, rocky pools, which tend to concentrate this seaweed [7,11], are sites sensitive to the increase of compounds released by retained *A. armata*. This release may ultimately present adverse effects for other organisms such as seaweed, vertebrates or invertebrates, leading to severe consequences for coastal ecosystems [7,12,13]. To investigate potential ecological impairments caused by these compounds, key species in the structure and functioning of coastal ecosystems were used: the common prawn *Palaemon elegans* and the gastropod *Gibbula umbilicalis*. These organisms inhabit the upper intertidal zone on rocky shores where *A. armata* is often found attached to the substrate, or unattached (drifting), and releasing its chemical exudates. In this work, the assessment of *A. armata* exudate effects on these marine invertebrates was performed by addressing survival and sublethal effects through behavioural and biochemical energy-related responses.

Feeding activity is a sensitive tool to assess the impact of contaminants at concentrations far below lethal levels, which is often related to a decrease of fitness and capacity to respond to contaminants and also often reflects alterations in movement capability, and that will reduce energetic input and metabolism [14,15,16,17]. Enzymes involved in energy production have been frequently used as biomarkers to assess the effects of stressors, since exposed organisms usually need additional energy to maintain physiological/biochemical functions [18,19,20]. Thus, biomarkers such as total content in energy reserves, lactate dehydrogenase (LDH) and electron transport system (ETS) activities may provide valuable information on the physiological status of the studied organisms. The purpose of this research was to address the effects of *A. armata* exudated secondary metabolites on the survival, behaviour, and energetic metabolism of two marine invertebrates inhabiting rock pools, providing further ecotoxicological insight into the invasive strategy of this seaweed.

## 2. Results

### 2.1. Survival

**Figure 1:**
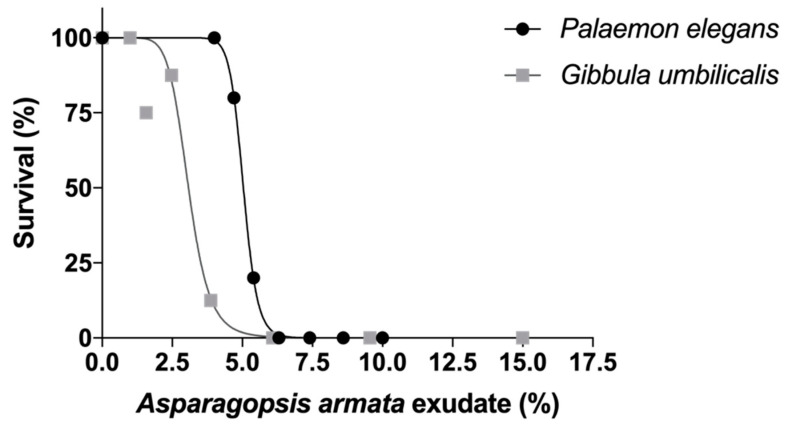
Survival rate of two marine invertebrates after 96 h exposure to *Asparagopsis armata* exudate. Black circle: *Palaemon elegans*; grey square: *Gibbula umbilicalis*.

Acute toxicity tests revealed that *A. armata* exudate affects both species, with *P. elegans* being more tolerant than *G. umbilicalis* with significantly higher LC_50_ (F_2,100_ = 53.03, *p* < 0.001) (Figure 1). *Gibbula umbilicalis* has a 96 h LC_50_ [95% CI] of 2.79% [1.66–4.69] and *P. elegans* a 96 h LC_50_ [95% CI] of 5.04% [4.84–5.25].

### 2.2. Behavioural Responses—Feeding Activity

**Figure 2:**
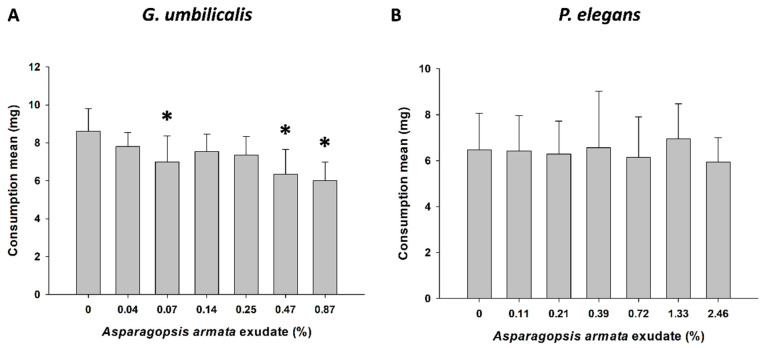
Feeding activity of *Gibbula umbilicalis* (**A**) and *Palaemon elegans* (**B**) exposed to *Asparagopsis armata* exudate for 96 h. Results are expressed as mean values ± standard error (SE); Asterisks (*) indicate significant differences compared to the control treatment (0%).

Feeding activity was affected in *G. umbilicalis* (F_6,49_ = 5.304, *p* < 0.001; Figure 2A) when exposed to *A. armata* exudate at 0.07% (Dunnett’s *p* = 0.022), 0.47% (Dunnett’s *p* < 0.001), and 0.87% (Dunnett’s *p* < 0.001). No significant differences were found in *P. elegans* feeding activity (F_6,36_ = 0.178, *p* = 0.981; Figure 2B).

### 2.3. Energy Metabolism-Related Biomarkers

**Figure 3:**
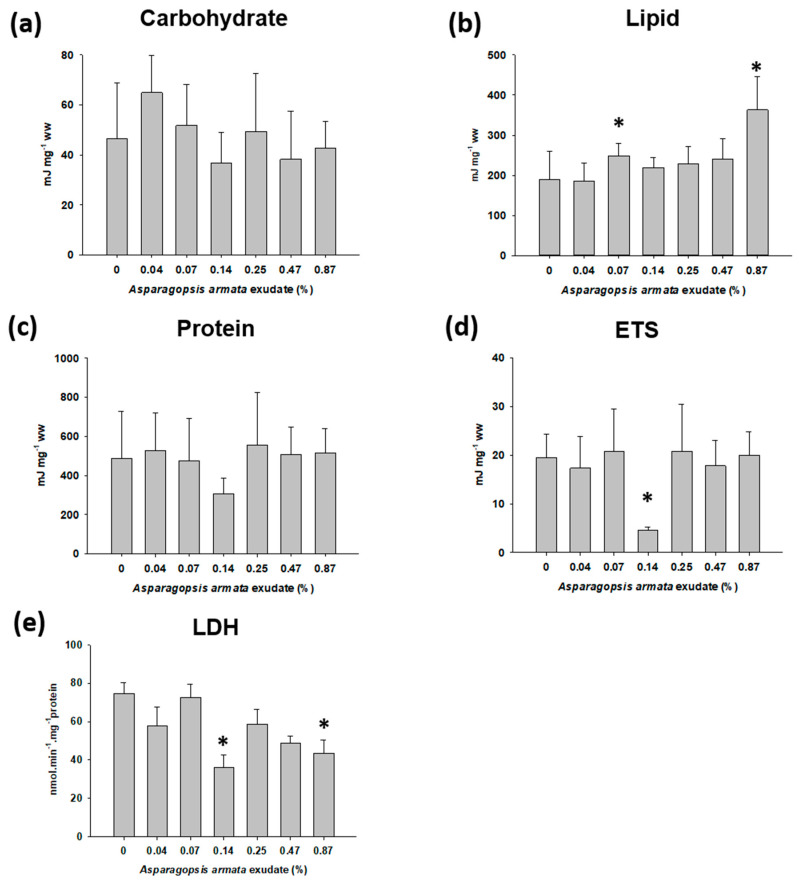
Energy-related parameters in *Gibbula umbilicalis* when exposed to *Asparagopsis armata* exudate for 168 h: (**a**) total carbohydrate content; (**b**) total lipid content; (**c**) total protein content; (**d**) electron transport system (ETS) activity; (**e**) lactate dehydrogenase (LDH) activity. Results are expressed as mean values ± SE; * Significant differences from the control (Dunnett’s or Dunn’s, *p* < 0.05).

Regarding *G. umbilicalis* exposure to *A. armata* exudate, no significant differences were observed in the carbohydrate and protein contents (Figure 3a,c). However, there was an increase in the accumulation of reserve lipids (F_6,47_ = 8.099; *p* < 0.001; Figure 3b) in 0.07% (Dunnett’s *p* = 0.031) and 0.87% (Dunnett’s *p* < 0.001) exudate exposures. ETS activity showed a significant decrease at 0.14% treatment (H_6_ = 19.784, Dunn’s *p* = 0.003; Figure 3d) whereas LDH was also significantly inhibited (F_6,44_ = 4.041, *p* = 0.003; Figure 3e) at 0.14% (Dunnett’s *p* = 0.002) and 0.87% (Dunnett’s *p* = 0.022).

**Figure 4:**
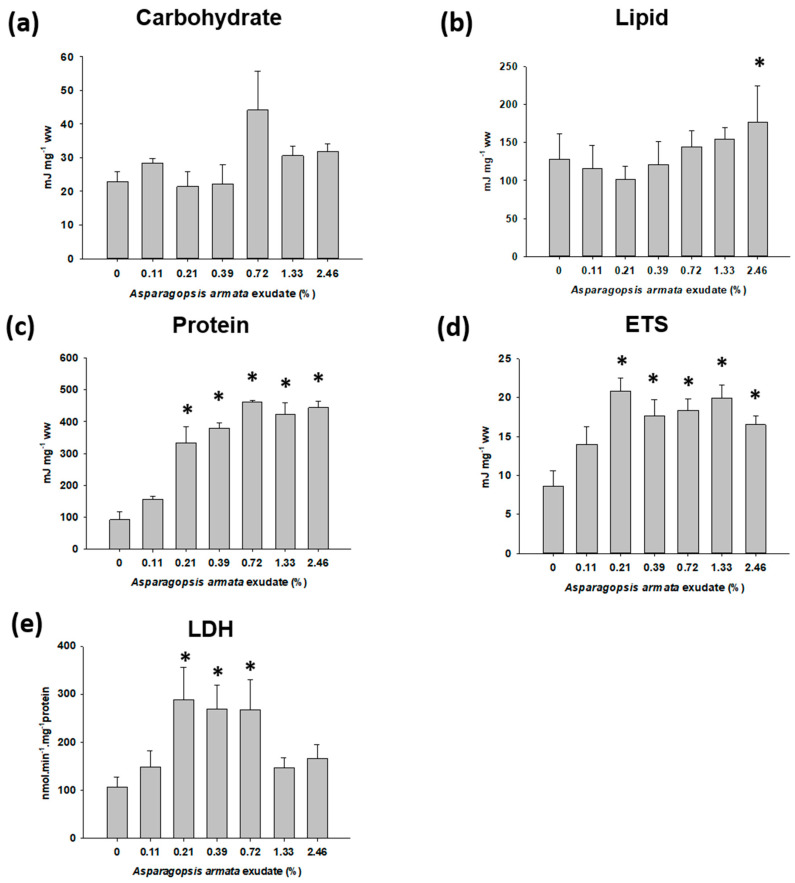
Energy related parameters in *Palaemon elegans* when exposed to *Asparagopsis armata* exudate for 168 h: (**a**) total carbohydrate content; (**b**) total lipid content; (**c**) total protein content; (**d**) electron transport system (ETS) activity; (**e**) lactate dehydrogenase (LDH) activity. Results are expressed as mean values ± SE; * Significant differences from the control (Dunnett’s or Dunn’s, *p* < 0.05).

As for the energy reserves measured in the muscle tissue of *P. elegans*, no effects were observed for carbohydrates (H_6_ = 9.431, *p* = 0.151) after exudate exposure (Figure 4a) but there was an increase in total lipids (F_6,44_ = 5.580, *p* < 0.001; Figure 4b) at the highest tested concentration (Dunnett’s *p* = 0.020). Mean protein levels were also significantly increased at all concentrations (F_6,38_ = 32.667, *p* < 0.001; Figure 4c) except at the lowest 0.11% (Dunnett’s *p* = 0.272). ETS followed the same pattern as protein accumulation, with an increase in activity for concentrations equal to or higher than 0.21% of exudate (F_6,45_ = 5.757, *p* < 0.001; Figure 4d). LDH, on the other hand, only showed an increment in activity (F_6,46_ = 3.106, *p* = 0.012; Figure 4e) at the intermediary concentrations 0.21% (Dunnett’s *p* = 0.009) and 0.39% (Dunnett’s *p* = 0.014) and 0.72% (Dunnett’s *p* = 0.036).

## 3. Discussion

There are several studies addressing detrimental effects of seaweeds upon invertebrate communities, but very few address the toxicity of seaweed secondary metabolites, such as in the study of the effects of *Ulva* sp. exudate on the gastropods *Littorina littorea* and *L. obtusata* [21]. The present study shows the potential of *A. armata* exudates to affect marine invertebrates such as *G. umbilicalis* and *P. elegans*. The acute tests demonstrated that the exudate induces mortalities at very high dilutions from the initial seaweed exudate, with a 96 h LC_50_ of 2.93% for *G. umbilicalis* and 5.05% for *P. elegans*. Such low *A. armata* exudate tolerance in both invertebrates are of concern due to the well documented importance of these species to the functioning of rocky shore communities [22].

The comparison between 96 h dose-response curves revealed that *P. elegans* were more tolerant than *G. umbilicalis*, with a significantly higher LC_50_. The sensitivity of *G. umbilicalis* to this exudate is less obvious, as in the well-documented tolerance of this species to extreme environmental conditions [23] and even to other contaminants (organophosphate pesticides and metals) [14,24,25].

Alteration of normal behavioural patterns, such as feeding activity, may pose serious risks to the success of the community species that co-inhabit intertidal rock pools. Here, feeding activity was impaired by the seaweed exudate at sublethal concentrations, which agrees with the literature showing that behavioural endpoints are sensitive tools to evaluate sub-lethal effects of contaminants [26]. To the best of our knowledge, there are few examples of feeding inhibition by seaweed compounds. Sea urchin (*Lytechinus variegatus*) feeding was inhibited with caulerpenyne, an oxygenated sesquiterpene extracted from *Caulerpa prolifera*, and cymopol, a monoterpene-bromohydroquinone from *Cymopolia barbata* [27], both green invasive seaweeds. Other studies evidenced that halogenated monoterpenes, isolated from the red algae *Plocamium lepitophyllum*, inhibit food consumption by sea urchins and gastropods [28], and *A. armata* dichloromethane extracts have also revealed feeding deterrence activity [13].

Alteration in feeding is generally one of the first responses to environmental perturbations. Inhibition of feeding behaviour can result in reductions in energy assimilation, growth, reproduction, and survival [29,30]. In this work, the exudate derived from *A. armata* deterred the feeding of the marine gastropod *G. umbilicalis*, which is potentially due to the chemical defence characteristics of *A. armata*, documented in the literature as possessing compounds, mostly halogenated, with the primary function to deter herbivory [13,31,32]. Often, feeding inhibition derives from movement reduction due to toxic exposure, and consequently less capacity to find food and to process it [14], which may result in impacts on energy uptake and allocation, and ultimately have important ecological consequences [33]. The secondary metabolites (i.e., allelochemicals) produced by *A. armata* act as chemical defences against competitors and predators [34,35]. However, few studies have examined the effects of seaweed allelochemicals on both biochemical and behavioural responses, with most research focused on their defensive functions against herbivory. In this work, *A. armata* exudate not only interfered with the feeding behaviour of one of the species but also induced changes in several energy metabolism-related biochemical parameters of both invertebrates.

Regarding the effects on *G. umbilicalis*, *A. armata* induced a significant increase in total lipids, along with decreased activities of LDH and ETS. The primary source of energy is the carbohydrates followed by lipids and then proteins, and their mobilization is often used to counter toxic stress. Notwithstanding this, the high lipid content may be related to the fact that the invertebrates maintain the energy reserves as they alter their behaviour, decreasing their activity, and thus with less expenditure. In fact, the sea snail became less active, as seen in the proxy feeding at higher concentrations of exudate. This may ultimately lead to an energetic shift with less energy expenditure, despite the probable higher energetic demands for detoxification mechanisms. This trend has been reported in the literature for other compounds, as in the study of Verslycke and co-authors [36] with mysids, where a decrease in feeding was observed along with increasing lipid levels after exposure to chlorpyrifos.

LDH is an important glycolytic enzyme in biological systems and its activity is an indication of increased energy demand [37,38]. The significant decrease in LDH verified in the higher exudate concentrations, indicative of a reduction in anaerobic capacity, may reflect systemic toxicity impairing the organisms to fight the toxic stress. This decrease in general cellular metabolism is also in line with ETS results, where the decrease in the aerobic capacity is also suggested by the significant reduction of ETS in marine snails exposed to the medium concentration of 0.14%. This non-monotonic response to the exudate may derive from the complexity in the media and differentiated mechanisms of action of different individual compounds and their different concentrations in the mixture at a given exudate dilution, and constitutes an extra challenge to the interpretation of results of such nature.

In exposed *P. elegans* there was an increase in lipids and proteins along with the increase of ETS and LDH activities. The increase in lipids was similar as discussed previously for the sea snails, but for *P. elegans*, the exudate also exerted a significant increase of protein content. This increase may also reflect an induction in protein synthesis for detoxification processes and other defence mechanisms [39]. This is also supported by the metabolic reactions assessed which, contrary to the sea snails, indicate an enhancement of the cellular metabolism, with LDH and ETS activities being increased at the same concentrations. This indicates that the organisms are spending energy both anaerobically and aerobically to fight stress caused by *A. armata* exudate. This pattern indicates that the amount of energy available for growth, moulting, reproduction and other biological functions may be compromised [30]. It cannot be excluded that the steep increase of protein in *P. elegans* muscle may also be due to other factors rather than merely metabolic. The shrimps were not fed during the short 168 h exposure, and lesser locomotion (not addressed in this study) of exposed shrimps may translate into less energy expenditure and more accumulated protein.

These results indicate that organisms have different energy requirements to deal with the stress caused by the seaweed exudate. This comparative analysis may provide important insights into the heterogeneous effects of the *A. armata* exudate, driven by species-specific metabolic susceptibility patterns.

The present work was performed using exudates which to simulate natural conditions, demand that the seaweed is placed in seawater at a given ratio and defined conditions. This represents what is exudated by *A. armata* in nature—mostly bromoform and dibromoacetic acid, as in Paul et al. [32]. Ratios of 1:10 (the same as present stock solution) and over are commonly found in rock pools, which may remain enclosed from minutes to several hours during a tidal period. Other factors may also induce additional stress and compound release, as in the case of temperature or hydrodynamics [40]. Moreover, the exudates used here were prepared in the dark, and the production rate of volatile halogenated organic compounds (VHOCs) tends to decrease under dark conditions [41]. Despite the difficulties of benchmarking this stressor in the preparation of real scenario concentrations, the very high toxicity of this seaweed might not even reflect the worst-case scenario of exposure to exudates from this seaweed, especially in bloom events, in summer, in tide pools, where a body of water separates from the sea for hours during a tidal cycle, where seaweed concentrations are high and water dilution is diminutive.

## 4. Conclusions

The present study is an important step in the research of natural toxic exudates released into the environment and how they can affect the surrounding organisms and their mode of action in the invaded ecosystems. Although, as stated, this exudate contains a myriad of compounds, its toxicity is attributed mainly to the halogenated secondary metabolites produced and stored in vacuoles within the gland cells of *A. armata* (Burreson et al., 1976). The toxic effects recorded herein occurred at high dilutions of the prepared exudate. To better understand these chemical defences and the real impact in coastal environments, especially in more exposed tidal pools, further studies should be undertaken to determine the concentrations and variation in bioactive secondary metabolites, with respect to seaweed density and other biotic and abiotic factors. The impact on coastal environments may be more extensive compared to experiments in a controlled environment, with increased stress and production of secondary metabolites. Finally, the seaweed density can be higher, especially during seasonal seaweed stranding events, and other factors may affect secondary metabolite production in a high stress-burden ecosystem.

## 5. Materials and Methods

### 5.1. Test Organisms

The common prawn *Palaemon elegans* and the sea snail *Gibbula umbilicalis* were collected from Carreiro de Joannes, a rocky beach in Peniche, central Portugal (39°21′18.0″ N, 9°23′40.6″ W), with no known sources of chemical contamination. The organisms were maintained for 7 d in the laboratory in natural seawater at 20 ± 1 °C, with a 16:8 h (light:dark) photoperiod in aerated aquaria. Shrimps were fed mussels and snails fed *Ulva lactuca* until use. Prior to testing, organisms were kept fasting for 24 h.

### 5.2. Asparagopsis armata Collection and Preparation of Exudates

*Asparagopsis armata* was collected by scuba from the protected marine area around the Berlenga Island, Peniche, Portugal (39°25′03.0″ N, 9°30′23.6″ W). In the lab, after being cleaned and sorted, four aquaria with 5 kg of *A. armata* and 50 L of natural filtered seawater (through 0.45 µm cellulose acetate membrane filters) were left in the dark at 20 °C. After 12 h, the seaweed was removed, and the water from the different aquaria was pooled and sieved for bigger particles, followed by filtration through a 0.45 µm cellulose acetate membrane filter. The exudate was then kept in PET bottles at −20 °C until further use and represented the 100% stock concentration for all studies. This constituted the stock solution for all experiments and the 100% concentration.

### 5.3. Exposure Setup

All experiments were conducted in a climate-controlled room at 20 ± 1 °C, with a 16:8 h (light:dark), and experimental replicates consisted of glass vials with 60 mL and 750 mL exudate solution (or seawater in controls) for snails and shrimps, respectively, with one organism each. Flasks were covered with a plastic mesh to prevent organism escape and to assure constant submersion. An exudate stock solution vial was thawed daily and solutions were renewed every 24 h to avoid excreta accumulation and possible loss of volatile compounds. Exudate concentrations are presented as % of the exudate produced as described in Section 5.2.

#### 5.3.1. Survival

After a range-finding test, sea snails were exposed to increasing concentrations ranging from 1 to 15% of exudate (1; 1.57; 2.47; 3.87; 6.08; 9.55; and 15%), and shrimp from 4 to 10% of exudate (4; 4.66; 5.43; 6.32; 7.37; 8.58; and 10%). Exposures lasted 96 h and mortality was recorded daily. During exposures, no food was added. Eight and five replicates per treatment were used for sea snails and shrimps respectively, including a control treatment with filtered seawater only.

#### 5.3.2. Sublethal Exposure for Biomarker Analysis

Information on the lethal effects was used to establish maximum concentrations and conditions for each independent sublethal test, using half the LC_10_ as the highest concentrations tested. Sea snails were exposed to increasing concentrations of exudate ranging from 0.04 to 0.87% (0; 0.04; 0.07; 0.14; 0.25; 0.47; and 0.87%), and shrimp were exposed from 0.11 to 2.46% of exudate (0; 0.11; 0.21; 0.39; 0.72; 1.33; and 2.46%). Exposures lasted 168 h and 16 replicates per treatment were used for snails and 8 for shrimps, including a control treatment with filtered seawater only. At the end of the exposure period, the snail’s shell was broken with a vise, and soft tissues removed with forceps, weighed and kept on ice for operculum removal. Shrimps were sacrificed by decapitation and dissected. The abdominal muscle was rapidly isolated on ice and weighed. Tissues were maintained at −80 °C until further analysis.

#### 5.3.3. Sublethal Exposure for Feeding Inhibition Testing

For the feeding activity assay, concentrations of the exudate were the same as for the biomarker exposure, with 8 replicates per treatment for both invertebrates and exposed for 96 h. Organisms were fed with discs of *Ulva lactuca* with c.a. 10 cm^2^ previously dried at 60 °C for 48 h, weighed and re-hydrated just before adding to the medium (one disc per replicate). At the end of the 96 h exudate-exposure feeding test, the discs were rinsed, dried again, weighed, and the feeding was assessed by subtracting the algal final weight to its initial dry mass (mg).

### 5.4. Biomarkers Analysis

#### 5.4.1. Tissue Preparation

Snails were processed as pools of two individually exposed organisms. Each pool was considered as one biological replicate for the biomarker analysis (N = 8). For shrimps, the muscle tissue of each organism was processed individually and considered as one biological replicate (N = 8). The replicate tissues of each invertebrate species were homogenized in potassium phosphate buffer (0.1 M, pH 7.4) at a ratio (m:v) of 1:12 for *G. umbilicalis* and 1:10 for *P. elegans*. The homogenate was then separated into different microtubes to analyse total protein, carbohydrate, and lipid content. The remaining homogenate was separated into two fractions centrifuged respectively at 1000× *g* for 5 min (4 °C) for ETS measurement and 3000× *g* for 5 min (4 °C) for LDH measurement. All aliquots were stored at −80 °C until further analysis.

#### 5.4.2. Energy Reserves

Carbohydrate, lipid, and total protein contents were measured according to the approaches outlined by De Coen and Janssen [42,43]. The total carbohydrate content was determined in a reaction with phenol 5% and H_2_SO_4_ (95–97%), using glucose as standard and measuring absorbance at 490 nm [42]. Lipid content was determined according to Bligh and Dyer [44], using tripalmitin as standard and measuring absorbance at 400 nm. The total protein content was determined using the Bradford method [45], with bovine serum albumin as standard, measuring absorbance at 600 nm. Following De Coen and Janssen [42,43], all energy reserves were transformed into their energetic equivalents (39.5 kJ g^−1^ lipid, 24 kJ g^−1^ protein, 17.5 kJ g^−1^ glycogen).

#### 5.4.3. Energy Metabolism-Related Enzymes

Electron transport system (ETS) activity was determined following the method described by De Coen and Janssen [42]. The ETS activity was measured spectrophotometrically by adding NADPH solution and INT (p-iodonitrotetrazolium) to the sample and absorbance was read at 490 for 3 min. The oxygen consumption was then calculated using the stoichiometric relationship: 2 µmol of formazan formed = 1 µmol of oxygen consumed. The oxygen consumption rate was then converted into the energetic equivalent of 484 kJ mol^−1^ O_2_ for average carbohydrate, lipid, and protein consumption combinations [46].

The activity of LDH was measured following Vassault [47] with adaptations of Diamantino et al. (2001). The process is based on the efficiency of LDH to convert pyruvate to lactate, in the presence of NADH, which results in NADH oxidation and consequent decrease in absorbance. The absorbance was read at 340 nm for 5 min. A molar extinction coefficient of 6.3 × 10^3^ M cm^−1^ was used, and results were expressed as nmol min^−1^ mg protein^−1^.

### 5.5. Statistical Analysis

Significant differences between each treatment for biomarker analyses and behavioural parameters were studied using one-way analysis of variance (ANOVA) and differences to control were addressed by Dunnett’s post-hoc test. Normality was checked by the Shapiro–Wilk test and homoscedasticity by the Levene test. In the case of non-normally distributed data, the Kruskal–Wallis test was applied followed by Dunn’s post-hoc test. Statistical analyses for biochemical and behaviour endpoints were performed with the software SigmaPlot (Systat Software, San Jose, CA, USA) and LCs, correspondent 95% confidence intervals, and global fitting was undertaken using GraphPad Prism version 7 for Mac (GraphPad software, San Diego, CA, USA).
